# Prediction of outcomes in patients with metabolic dysfunction-associated steatotic liver disease based on initial measurements and subsequent changes in magnetic resonance elastography

**DOI:** 10.1007/s00535-023-02049-9

**Published:** 2023-10-16

**Authors:** Takashi Kobayashi, Michihiro Iwaki, Asako Nogami, Nobuyoshi Kawamura, Yasushi Honda, Yuji Ogawa, Kento Imajo, Masato Yoneda, Satoru Saito, Atsushi Nakajima

**Affiliations:** 1 Department of Gastroenterology and Hepatology, Yokohama City University Graduate School of Medicine, 3-9 Fukuura, Kanazawa-Ku, Yokohama, 236-0004 Japan; 2 Department of Gastroenterology, Shin-Yurigaoka General Hospital, Kawasaki, Japan; 3 Gastroenterology Division, National Hospital Organization Yokohama Medical Center, Yokohama, Japan

**Keywords:** Metabolic dysfunction-associated steatotic liver disease, Magnetic resonance elastography, Non-invasive test, Liver stiffness measurement

## Abstract

**Background:**

The prognosis of metabolic dysfunction-associated steatotic liver disease (MASLD) is strongly associated with liver fibrosis. We aimed to investigate whether liver stiffness measurement (LSM) and changes in LSM (ΔLSM) on magnetic resonance elastography (MRE) can predict clinical events in patients with MASLD.

**Methods:**

We included 405 patients with MASLD who underwent at least two MREs. The patients were divided into five groups corresponding to fibrosis stages (0–4) based on initial LSM and classified as progressors (ΔLSM ≥ 19%) or non-progressors (ΔLSM < 19%) based on the difference between the first and last LSM.

**Results:**

The mean follow-up period was 72.6 months, and the mean interval between MREs was 23.5 months. There were 52 (12.8%) progressors and 353 (87.2%) non-progressors. The initial LSM was significantly associated with the cumulative probabilities of decompensated cirrhosis, hepatocellular carcinoma (HCC), liver-related events, extrahepatic malignancies, and overall mortality but not with cardiovascular disease. Progressors had significantly higher hazard ratios (HRs) for decompensated cirrhosis, HCC, and liver-related events but not for extrahepatic malignancies, cardiovascular disease, or overall mortality. Among patients without cirrhosis, the HR for developing cirrhosis among progressors was 60.15. Progressors had a significantly higher risk of liver-related events, even in the low initial LSM (fibrosis stage 0–2) subgroups.

**Conclusions:**

Both initial LSM and ΔLSM can predict liver-related events in patients with MASLD, even for low initial LSM. This integrated assessment can allow more detailed risk stratification compared with single LSM assessments and identify high-risk patients with MASLD among those previously considered as low risk.

**Supplementary Information:**

The online version contains supplementary material available at 10.1007/s00535-023-02049-9.

## Introduction

Metabolic dysfunction-associated steatotic liver disease (MASLD), formerly known as non-alcoholic fatty liver disease, is the leading cause of chronic liver disease worldwide, with a prevalence of 25% in the general population [1–3]. The prognosis of patients with MASLD is most strongly associated with liver fibrosis [4–6]. Large prospective studies and meta-analyses have found that fibrosis stage ≥ 2 or ≥ 3 is associated with an increased incidence of liver-related and all-cause mortality [6, 8]. Accordingly, there is an urgent need to develop non-invasive tests that can predict the severity and prognosis of liver diseases in patients with MASLD.

Magnetic resonance elastography (MRE) has been established as the most accurate non-invasive diagnostic method for liver fibrosis; furthermore, MRE findings are currently used as endpoints in clinical trials [9–11]. Additionally, recent studies have attempted to improve the diagnostic capabilities of MRE by combining MRE values with clinical data. This has allowed the prediction of liver-related complications, including esophageal varices [12–14]. Recently, a study suggested that changes in MRE-assessed liver stiffness measurements (LSMs) over time could predict clinical outcomes in patients with MASLD [15]. However, this study included a small number of patients; moreover, the integrated assessment of baseline LSM values and their changes was not performed. Thus, the present study aimed to investigate whether the combined assessment of LSM at baseline and its changes during the follow-up period could accurately predict liver-related and extrahepatic events in a larger cohort of Asian patients with MASLD.

## Methods

### Participants and definitions

This retrospective cohort study included patients with MASLD who underwent at least two MRE measurements for liver fibrosis progression at Yokohama City University between January 2012 and August 2022. The study protocols were approved by the Institutional Review Board of Yokohama City University (B190200023), and the study was conducted according to the Declarations of Helsinki and Istanbul. Written informed consent for participation in the study was obtained from each patient.

MASLD was diagnosed based on the presence of fatty liver as observed on abdominal ultrasound or histological evaluation. The current and past daily alcohol intakes were < 30 g/day and < 20 g/day in all male and female patients, respectively. Details regarding alcohol consumption were obtained by the treating physicians and confirmed by close family members. None of the patients received any medications that could cause steatotic liver disease. All patients met at least one of the following cardiometabolic criteria in the MASLD definition: (1) BMI ≥ 23 kg/m^2^, waist circumference > 94 cm (male), and waist circumference > 80 cm (female); (2) fasting serum glucose level ≥ 100 mg/dL or 2-h post-load glucose level ≥ 140 mg/dL, or HbA1c level ≥ 5.7%, or type 2 diabetes, or ongoing treatment for type 2 diabetes; (3) blood pressure ≥ 130/85 or ongoing specific antihypertensive drug treatment; (4) plasma triglyceride level ≥ 150 mg/dL or ongoing lipid-lowering treatment; and (5) plasma HDL-cholesterol level ≤ 40 mg/dL (male), level ≤ 1.3 mg/dL (female), or ongoing lipid-lowering treatment. In this study, a BMI cutoff of 23 kg/m^2^ was used because all patients were Japanese [1]. Patients with the following disorders were excluded: secondary causes of steatotic liver disease, drug-induced liver disease, alcoholic liver disease, viral hepatitis, autoimmune hepatitis, primary biliary cholangitis, alpha 1-antitrypsin deficiency, hemochromatosis, Wilson’s disease, and biliary obstruction. To avoid false assessments of LSM, patients were examined while fasting. Data regarding age, sex, body mass index, presence of diabetes mellitus, and hypertension at the time of initial MRE were collected. Magnetic resonance imaging-based proton density fat fraction was measured simultaneously with MRE. Furthermore, laboratory test results such as aspartate aminotransferase (AST), alanine aminotransferase (ALT), platelets, and triglyceride levels were collected within 30 days before or after MRE. The Fibrosis-4 index was calculated as follows: age (years) × AST (*U*/*L*)/(platelets [10^9^/*L*] × ALT^1/2^ [*U*/*L*]) [16].

Cirrhosis was defined as a histopathological diagnosis of fibrosis stage 4 based on the NASH Clinical Research Network Scoring System. In addition, cirrhosis was indicated by the presence of both morphological changes consistent with cirrhosis (surface irregularities, caudal lobe hypertrophy, right lobe atrophy) and findings of portal hypertension (splenomegaly or portosystemic shunting, esophageal varices, portal hypertensive gastropathy). Liver morphological change was evaluated by computed tomography (CT), MRI or ultrasound, and portal hypertension was assessed by either CT, MRI, or gastrointestinal endoscopy. Decompensated cirrhosis was defined by the presence of at least one of the following findings: gastroesophageal variceal hemorrhage, ascites, hepatic encephalopathy, or jaundice. Gastroesophageal variceal bleeding was confirmed by upper endoscopy, while ascites was confirmed by imaging. If an outpatient had ascites on imaging studies, the date of the imaging study was defined as the date of onset of decompensated cirrhosis. Cardiovascular disease was defined as the first occurrence of angina pectoris or myocardial infarction; further, liver-related events were defined as the first occurrence of hepatocellular carcinoma (HCC) or decompensated cirrhosis.

The clinical outcomes of all patients were examined. An overall clinical event was defined as the first occurrence of a liver-related event, cardiovascular disease, extrahepatic malignancy, or all-cause mortality.

### MRE

Each patient underwent MRE using a 3.0-T MR system (GE Medical Systems, Milwaukee, WI, USA) after fasting for at least 4 h. MRE measurements were performed by an experienced hepatologist or radiologist who was blinded to information regarding clinical data and disease course as previously reported [17]. Regions of interest were drawn on each section of MRE images to only include the parenchyma of the right lobe while avoiding the liver edges and large blood vessels. The mean of the measurements in four slices was calculated for analysis. Given the heterogenous progression of liver fibrosis, this method was used to allow assessment of liver fibrosis in a larger area of the right lobe [17].

### Statistical analysis

Patients’ physical data and MRI measurements are presented as means, standard deviations, and percentages. In the analysis of initial LSM, patients were divided into five groups: Groups 0, 1, 2, 3, and 4 that corresponded to fibrosis stages 0–4 and an initial LSM < 2.61, 2.61–2.96, 2.97–3.61, 3.62–4.69, and > 4.69 kPa, respectively [10]. MASLD progression was assessed based on the difference between the two LSMs (ΔLSM) with MRE. According to the Quantitative Imaging Biomarkers Alliance consensus, ΔLSM > 19% was considered true and significant; accordingly, this value was used as the cut-point for defining progression or regression [18–20].

The follow-up date was defined as the period from the first MRE date to the date of the occurrence of a clinical outcome or the date of the last confirmed survival. For serial MRE analysis, only events that occurred after the second MRE and for the first time were recorded. The following clinical events were recorded: development of liver cirrhosis, decompensated cirrhosis, HCC, extrahepatic malignancies, cardiovascular diseases, and death. Liver-related events included HCC and decompensated cirrhosis.

The cumulative probability of liver-related events according to the baseline LSM or ΔLSM is expressed as a Kaplan–Meier survival curve. The probabilities were compared for statistical significance using the log-rank test. All statistical analyses were performed using the JMP Pro 15 software package (SAS Institute, Cary, NC, USA). Statistical significance was set at a *p* value < 0.05.

## Results

### Baseline characteristics

A total of 405 patients with MASLD underwent serial MREs. Table 1 summarizes the characteristics of the included patients. The mean age was 58.5 ± 2.5 years, and 219 (54.1%) patients were female. The mean initial LSM was 3.13 ± 0.94 kPa. The number of patients in each group was as follows: Group 0, 155 (38.3%); Group 1, 58 (14.3%); Group 2, 83 (20.5%), Group 3, 62 (5.3%); and Group 4, 47 (11.6%). The mean interval between consecutive MREs was 23.5 ± 0.8 months, and the mean interval from the initial MRE to the end of follow-up was 72.6 ± 25.8 months.

**Table 1:** Patient characteristics (*N* = 405)

Characteristic	Value
Age, mean (SD)	58.5 (2.5)
Sex, female, *n* (%)	219 (54.1%)
BMI, mean (SD)	29.9 (25.9)
Diabetes, *n* (%)	217 (53.5%)
Hypertension, *n* (%)	218 (53.7%)
AST, mean (SD), IU/L	43.3 (27.3)
ALT, mean (SD), IU/L	55.4 (41.5)
AST/ALT, mean (SD)	0.91 (0.38)
Platelets, mean (SD), × 10^9^/L	219 (72)
Triglyceride, mean (SD), mg/dl	173 (133)
FIB‐4, mean (SD)	2.17 (2.84)
Initial LSM, mean (SD), kPa	3.29 (1.40)
Group 0, equivalent to fibrosis stage 0 (< 2.61 kPa), *n* (%)	155 (38.3%)
Group 1, equivalent to fibrosis stage 1 (2.61–2.96 kPa), *n* (%)	58 (14.3%)
Group 2, equivalent to fibrosis stage 2 (2.97–3.61 kPa), *n* (%)	83 (20.5%)
Group 3, equivalent to fibrosis stage 3 (3.62–4.69 kPa), *n* (%)	62 (15.3%)
Group 4, equivalent to fibrosis stage 4 (> 4.69 kPa), *n* (%)	47 (11.6%)
Last LSM, mean (SD), kPa	3.16 (1.59)
Initial MRI-PDFF, mean (SD), %	13.4 (7.9)
Last MRI-PDFF, mean (SD), %	11.9 (7.1)

ALT, alanine aminotransferase; AST, aspartate aminotransferase; BMI, body mass index; FIB‐4, Fibrosis‐4 index; LSM, liver stiffness measurement; MRI-PDFF, magnetic resonance imaging-based proton density fat fraction; SD, standard deviation

### Changes in LSM

**Fig. 1:**
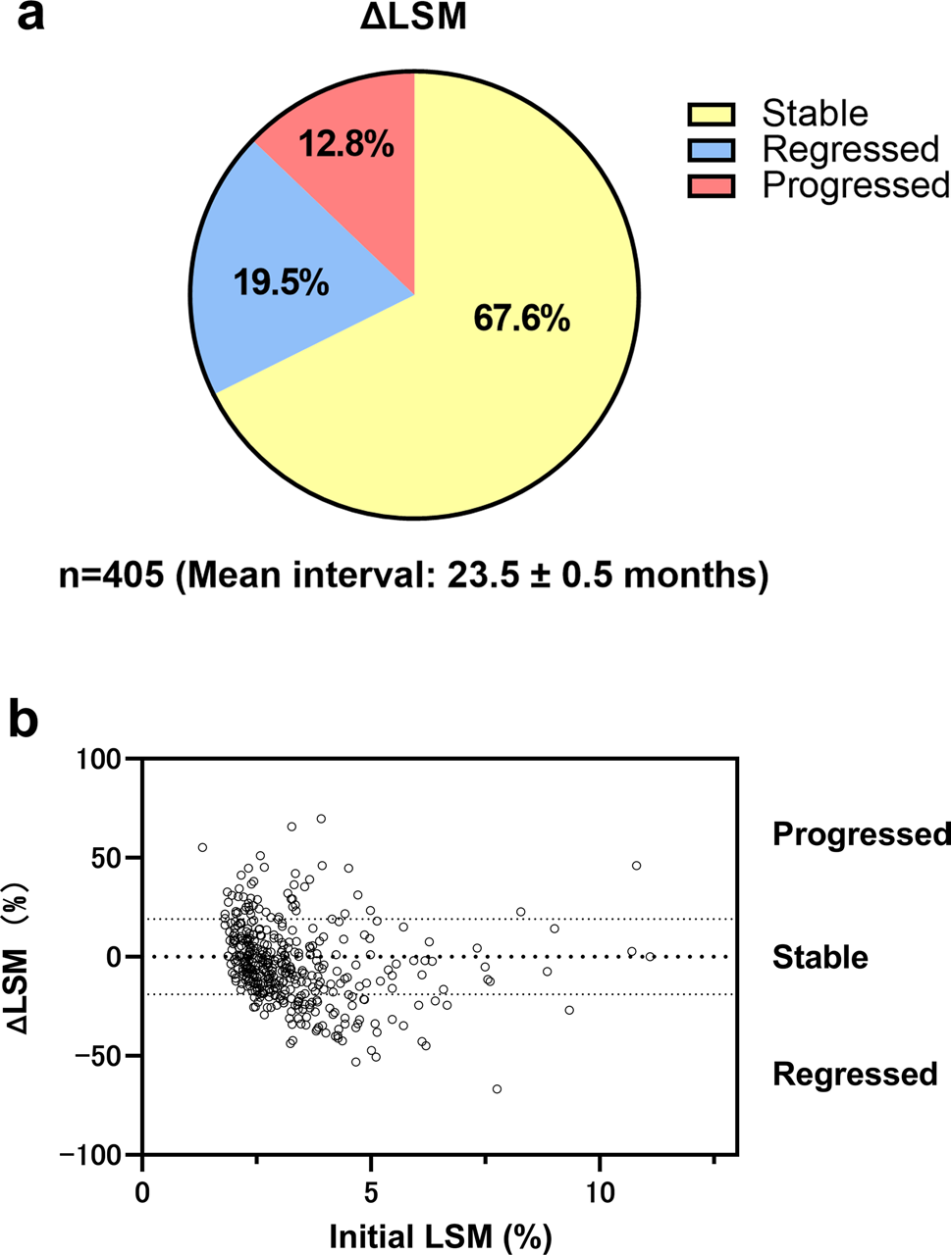
**a** Distribution of LSM changes. In serial MRE measurements in patients with MASLD, LSM regressed in 79 (19.5%) patients, remained unchanged in 274 (67.7%) patients, and progressed in 52 (12.8%) patients. The mean interval between the two MRE measurements was 23.51 ± 0.47 months. **b** Scatterplot exhibiting the distribution of patients divided into three groups: progression, stable, and regression. MRE, magnetic resonance elastography; LSM, liver stiffness measurements by magnetic resonance elastography; ΔLSM, difference between two LSMs

During the interval between serial MRE measurements, MASLD regressed in 79 patients (19.5%), remained unchanged in 274 patients (67.7%), and progressed in 52 patients (12.8%) (Fig. 1a). As demonstrated in Fig. 1b, progressors with high ΔLSM values were observed regardless of the initial LSM value.

### Clinical outcomes

Among the 405 patients, 11 (2.71%) died during the follow-up period. Furthermore, 17 (4.20%) patients developed a total of 18 liver-related events. HCC (*n* = 8) was the most common liver-related event, followed by ruptured gastroesophageal varices (*n* = 6). Extrahepatic malignancies occurred in 22 patients (5.43%), with colorectal cancer (*n* = 7) being the most common malignancy. Supplementary Table 1 presents details regarding extrahepatic malignancies. Cardiovascular diseases occurred in only six patients (1.48%). Among the 386 patients without clinical cirrhosis at the time of the initial MRE, 11 (2.8%) developed cirrhosis during the study period.

### Initial LSM as a predictor of clinical outcomes

**Fig. 2:**
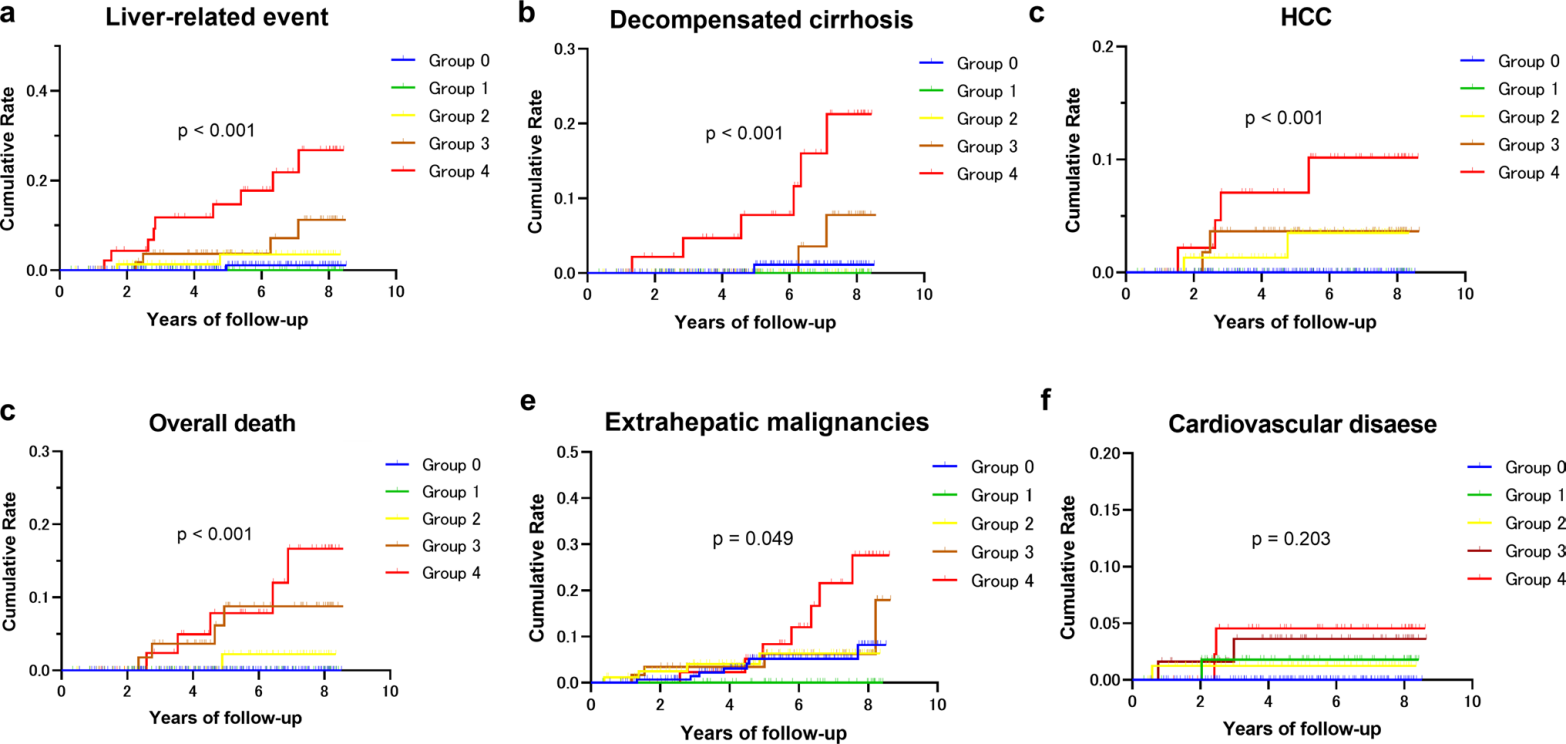
Cumulative incidence of clinical events based on the initial LSM. Patients were divided into five groups (Groups 0–4) corresponding to each fibrosis stage (fibrosis stages 0–4) according to the initial LSM. **a** Liver-related events; **b** decompensated cirrhosis; **c** hepatocellular carcinoma; **d** all-cause mortality; **e** extrahepatic malignancies; **f** cardiovascular disease. LSM: liver stiffness measurement by magnetic resonance elastography

Table 2 and Fig. 2 show the cumulative probabilities obtained from the Kaplan–Meier analysis of liver-related events, decompensated cirrhosis, HCC, overall mortality, extrahepatic malignancies, and cardiovascular disease, stratified by group based on the initial LSM. Within an observation period of 72.6 ± 25.8 months, there were significant among-group differences in the cumulative incidence of liver-related events (*p* < 0.001), decompensated cirrhosis (*p* < 0.001), HCC (*p* < 0.001), overall mortality (*p* < 0.001), and extrahepatic malignancies (*p* = 0.049) according to the log-rank test; however, there was no significant among-group difference in the incidence of cardiovascular disease (*p* = 0.203). Additionally, there was a significant among-group difference in the cumulative incidence of overall clinical events (*p* < 0.001) (Table 2 and Supplementary Fig. 1a).

**Table 2:** Cumulative probability of outcomes according to the initial LSM groups corresponding to the fibrosis stages (*N* = 405)

Event	Initial LSM group^a^	Events, *n*	Cumulative, %^b^	*p*
Liver-related events	0	1/155	0.6	< 0.001
	1	0/58	0.0	
	2	2/83	2.4	
	3	5/62	8.1	
	4	9/47	19.1	
Decompensated cirrhosis	0	1/155	0.6	< 0.001
	1	0/58	0.0	
	2	0/83	0.0	
	3	3/62	4.8	
	4	6/47	12.8	
Hepatocellular carcinoma	0	0/155	0.0	< 0.001
	1	0/58	0.0	
	2	2/83	2.4	
	3	2/62	3.2	
	4	4/47	8.5	
Overall death	0	0/155	0.0	< 0.001
	1	0/58	0.0	
	2	1/83	1.2	
	3	5/62	8.1	
	4	5/47	10.6	
Extrahepatic malignancies	0	7/155	4.5	0.048
	1	0/58	0.0	
	2	0/83	4.8	
	3	4/62	6.5	
	4	7/47	14.9	
Cardiovascular disease	0	0/155	0.0	0.203
	1	1/58	0.0	
	2	1/83	1.2	
	3	1/62	1.6	
	4	2/47	4.3	
Overall events	0	9/155	5.8	< 0.001
	1	1/58	1.7	
	2	7/83	8.4	
	3	12/62	19.4	
	4	17/47	36.2	

^a^The initial LSM group corresponds to liver fibrosis stages 0–4; ^b^Estimated using Kaplan–Meier analysis

LSM, liver stiffness measurement

As demonstrated in Supplementary Table 2, the HRs of liver-related events for Groups 3 and 4 vs. Group 0 were 10.48 (95% confidence interval [CI]: 1.55–70.68; *p* = 0.006) and 37.01 (95% CI 8.67–157.9; *p* < 0.001), respectively. Additionally, the respective HRs of Groups 3 and 4 vs. Group 0 were as follows: 4.81 (95% CI 0.42–54.28; *p* = 0.203) and 24.84 (95% CI 4.51–137.0; *p* < 0.001) for decompensated cirrhosis; 34.53 (95% CI 1.59–750.3; *p* = 0.024) and 60.29 (95% CI 6.15–591.1; *p* < 0.001) for HCC; 33.02 (95% CI 3.78–288.9; *p* = 0.003) and 45.33 (95% CI 6.17–333.0; *p* < 0.001) for overall death; 1.24 (95% CI 0.34–4.51; *p* = 0.744) and 3.66 (95% CI 1.10–12.19; *p* = 0.035) for extrahepatic malignancies; and 35.42 (95% CI 1.62–774.1; *p* = 0.024) and 64.88 (95% CI 2.52–1668; *p* = 0.012) for cardiovascular disease.

### ΔLSM as a predictor of clinical outcomes

**Fig. 3:**
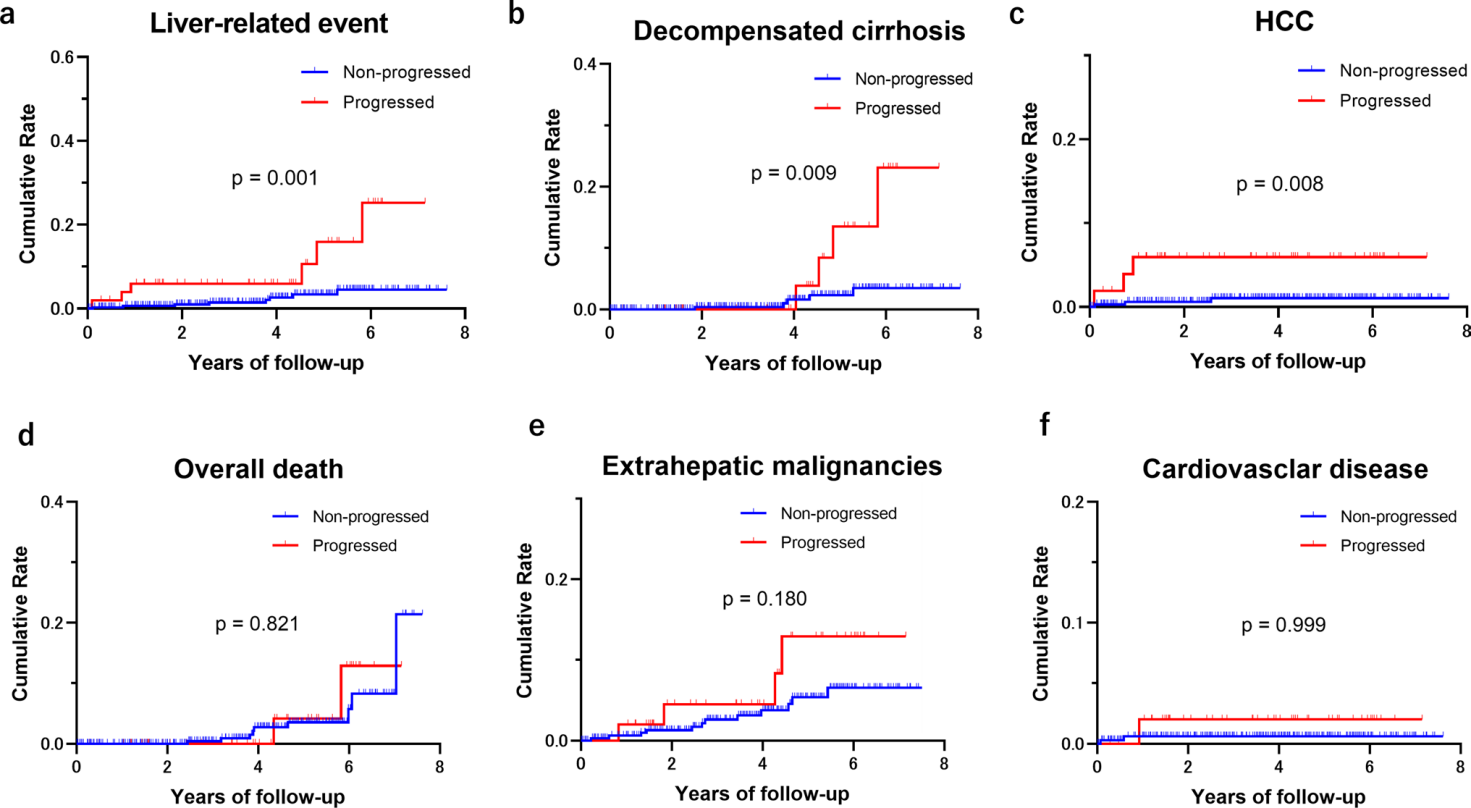
Cumulative incidence of clinical events in progressors vs. non-progressors (stable/regression) based on ΔLSM. **a** Liver-related events; **b** decompensated cirrhosis; **c** hepatocellular carcinoma; **d** all-cause mortality; **e** extrahepatic malignancies; **f** cardiovascular disease. ΔLSM: difference between two liver stiffness measurements by magnetic resonance elastography

The cumulative incidence of clinical events among progressors and non-progressors (stable/regressors) is demonstrated in Supplementary Table 3. The cumulative incidence of liver-related events, including decompensated cirrhosis and HCC, estimated by Kaplan–Meier curves was significantly higher in progressors than in non-progressors (11.5% [6/52 patients] vs. 2.3% [8/353 patients], *p* = 0.001, log-rank test) (Fig. 3a). Based on classification according to events, the cumulative incidence of decompensated cirrhosis and HCC was significantly higher in progressors than in non-progressors (decompensated cirrhosis (7.7% [4/52 patients] vs. 1.4% [5/353 patients], *p* = 0.009); HCC (5.8% [3/52 patients] vs. 0.8% [3/353 patients], *p* = 0.008) (Fig. 3b, c). Contrastingly, the cumulative probabilities of all-cause mortality (*p* = 0.820), extrahepatic malignancies (*p* = 0.180), and cardiovascular events (*p* = 0.317) did not significantly differ between progressors and non-progressors (Fig. 3d–f and Supplementary Table 3).

Furthermore, as shown in Table 3, compared with non-progressors, progressors had a significantly higher risk of liver-related events (HR: 12.79, 95% CI 2.74–59.64; *p* = 0.012), decompensated cirrhosis (HR: 12.08, 95% CI 1.85–78.73; *p* = 0.009), HCC (HR: 25.02, 95% CI 2.35–267; *p* = 0.08), and overall clinical events (HR: 3.72, 95% CI 1.41–9.84; *p* < 0.01) (Supplementary Fig. 1b and Table 3). Alternatively, the HRs for progressors vs. non-progressors were as follows: 1.21 (95% CI 0.24–6.15; *p* = 0.820) for all-cause mortality, 2.66 (95% CI 0.64–11.17; *p* = 0.180) for extrahepatic malignancies, and 5.40 (95% CI 0.20–247; *p* = 0.317) for cardiovascular diseases (Table 3).

**Table 3:** Relative risk of clinical outcomes in progressors compared with non-progressors based on serial magnetic resonance elastography

Event	HR	95% CI	*p*
*N* = 405			
Liver-related event	12.79	2.74–59.6	0.001
Decompensated cirrhosis	12.08	1.85–78.7	0.009
HCC	25.02	2.35–267	0.008
Overall death	1.21	0.24–6.15	0.821
Extrahepatic malignancies	2.67	0.64–11.2	0.180
Cardiovascular events	5.40	0.20–147	0.317
Overall events	3.72	1.41–9.84	0.008
*N* = 386			
New onset of cirrhosis	60.15	11.0–329	< 0.001

Patients were classified as progressors (ΔLSM ≥ 19%) and non-progressors (ΔLSM < 19%) according to the difference between the first and last liver stiffness measurement (ΔLSM)

CI, confidence interval; HR, hazard ratio

Since LSM changes in patients with cirrhosis may be small, we evaluated the correlation between ΔLSM and clinical events in a subgroup of patients without pathologic or clinical cirrhosis at the time of the second MRE (*n* = 386). Even in this subgroup, progressors were at a significant risk of liver-related events (HR: 10.91, 95% CI 1.29–92.18; *p* = 0.028) and HCC (HR: 24.60, 95% CI 1.36–443.7; *p* = 0.030) compared with non-progressors; moreover, they showed a non-significant trend toward a higher risk of decompensated cirrhosis (HR: 15.09, 95% CI 0.98–232.5; *p* = 0.052). Contrastingly, there was no significant difference in the risk of all-cause mortality (HR: 1.85, 95% CI 0.64–5.37; *p* = 0.258), extrahepatic malignancies (HR: 1.71, 95% CI 0.39–7.52; *p* = 0.480), or cardiovascular disease (HR: 1.57, 95% CI 0.13–19.10; *p* = 0.127) between progressors and non-progressors (Supplementary Fig. 2).

### ΔLSM predicts the progression to cirrhosis in patients without cirrhosis

Among patients without pathologic or clinical cirrhosis at the time of the second MRE (*n* = 386), 50 (13.0%) progressed over serial MRE measurements, whereas 336 (87.0%) had stable or regressed LSM. Within a follow-up period of 43.9 ± 39.4 months after the second MRE, progressors had a significantly higher incidence of cirrhosis than non-progressors (7/50 patients [14.0%] vs. 2/336 patients [1.2%]) (Supplementary Fig. 3). Compared with non-progressors, progressors had a significantly higher risk of developing cirrhosis (HR: 60.15, 95% CI 11.0–329; *p* < 0.001) (Supplementary Fig. 3, Table 3).

### ΔLSM as a predictor of clinical outcomes in patients with a low initial LSM

**Fig. 4:**
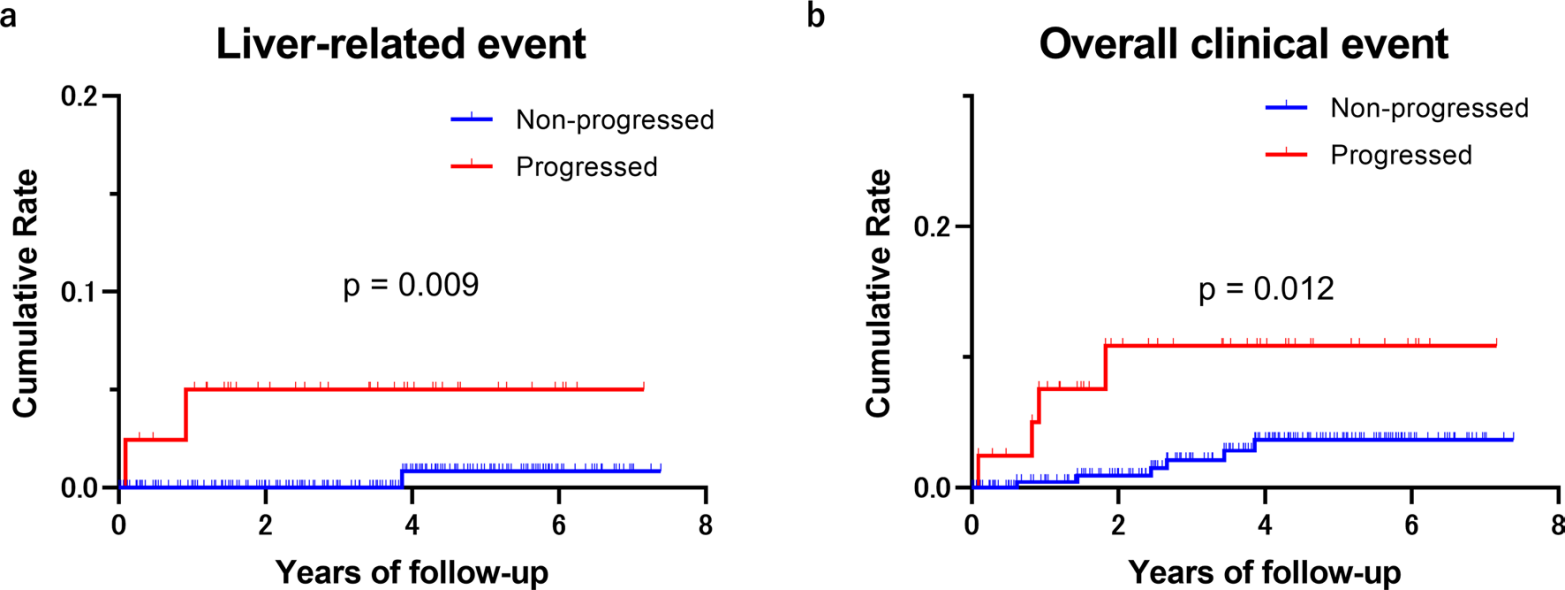
Cumulative incidence of clinical events in progressors versus non-progressors (stable/regressive) based on ΔLSM in patients with MASLD with a low initial LSM corresponding to fibrosis stage ≤ 2 (*N* = 296). **a** Liver-related events; **b** Overall clinical events. LSM: liver stiffness measurement by magnetic resonance elastography; ΔLSM: difference between two liver stiffness measurements by magnetic resonance elastography

In a subgroup of patients with an initial LSM of < 3.62 kPa (*n* = 296), which corresponds to fibrosis stage 0–2, indicating a low risk of clinical events, we evaluated the rate of clinical events in progressors and non-progressors. In this subgroup, the cumulative incidence of liver-related events was significantly higher in progressors than in non-progressors (4.9% [2/41] vs. 0.4% [1/255], *p* = 0.009) (Fig. 4a and Supplementary Table 4). Additionally, the cumulative incidence of all clinical events was significantly higher in progressors than in non-progressors (14.6% [6/41] vs. 1.6% [4/255], *p* = 0.012) (Fig. 4b and Supplementary Tables 4 and 5). The HRs for progressors vs. non-progressors for liver-related events and all events were 77.45 (95% CI 2.99–2006; *p* = 0.009) and 10.32 (95% CI 1.656–64.37; *p* = 0.012), respectively (Supplementary Table 5).

## Discussion

We analyzed baseline LSM and changes in LSM and their association with subsequent clinical events in patients with MASLD. Our findings indicated that initial LSM was significantly associated with decompensated cirrhosis, HCC, all liver-related events, extrahepatic malignancies, and all-cause mortality but not with cardiovascular disease. Contrastingly, ΔLSM was significantly associated with decompensated cirrhosis, HCC, and all liver-related events but not with extrahepatic malignancies, cardiovascular events, or all-cause mortality. Furthermore, ΔLSM could predict the development of cirrhosis in patients without cirrhosis. Moreover, even among patients with low initial LSM who would be assumed to be without advanced fibrosis and have good prognosis, the risk of liver-related and overall clinical events was significantly higher in progressors than in non-progressors.

In patients with MASLD, liver fibrosis has been widely reported to be strongly associated with mortality and liver-related events [4–7, 21]. Moreover, several non-invasive tests for diagnosing liver fibrosis have shown a predictive capability for clinical events, which are comparable with that of liver biopsy, in patients with MASLD [22]. LSM utilizing MRE, which is the most accurate non-invasive tests for liver fibrosis, has been reported to be associated with the incidence of liver-related events in patients with chronic liver disease, including MASLD [13, 23]. A recent study reported that changes in serial LSMs could predict the incidence of liver-related events [15].

In our study, we analyzed both baseline LSM and changes in LSM, which were both significantly associated with the incidence of liver-related events, including HCC. Moreover, we found that ΔLSM could significantly predict the development of cirrhosis in patients without cirrhosis. Furthermore, even among patients with a low initial LSM, which is generally considered indicative of good prognosis, progressors had a higher incidence of liver-related events than non-progressors. Thus, an integrated assessment of single and serial LSMs may allow improved stratification of risk. Specifically, not only patients with a high initial LSM, but also those with an increase in LSM by > 19% during follow-up should be carefully monitored as high-risk patients. Moreover, evaluation of ΔLSM with repeated MREs, rather than just a single MRE, may allow detection of latent high-risk patients, especially in patients with a low initial LSM. Additionally, among patients with a high initial LSM, those with a high ΔLSM may require even closer monitoring as very high-risk patients.

There were some notable differences between the predictive utility of the initial LSM and ΔLSM for clinical events. Single measurements were significantly associated with decompensated cirrhosis, HCC, all liver-related events, extrahepatic malignancies, and all-cause mortality but not with cardiovascular disease. Contrastingly, ΔLSM was only associated with decompensated cirrhosis, HCC, and all liver-related events. Although the reasons underlying this difference are unclear, our findings indicated that high LSM values are associated not only with liver-related events but also with all-cause mortality and extrahepatic malignancies. A recent large-scale meta-analysis reported an association between high LSM values and all-cause mortality [13]. There have been inconsistent reports regarding the relationship between extrahepatic malignancies and liver stiffness or fibrosis. However, a Swedish large nationwide cohort study found that histopathological progression, including fibrosis, was associated with increased mortality from extrahepatic malignancies [24]. Accordingly, patients with rapidly increasing liver stiffness, especially those with progressive conditions, i.e., those with a high disease activity and progressive liver histopathology, may be particularly at risk of liver-related events.

MRE has been reported to be the most accurate biomarker for assessing liver fibrosis [25, 26]. However, unlike histopathological evaluation, LSM cannot detect inflammation or ballooning, which makes it unsuitable for assessing disease activity. Our findings indicated that serial LSMs allow more dynamic evaluation of disease changes and help compensate for the disadvantages of MRE. Our findings support the use of MRE as a non-invasive test that may be used to overcome the disadvantages of liver biopsy and demonstrate its utility in providing a dynamic biomarker in new drug development. Additionally, unlike liver biopsy, MRE can be performed repeatedly, and future studies using MRE may reveal factors that influence the progression of liver fibrosis.

Vibration-controlled transient elastography (VCTE) is another technique for measuring liver stiffness and has demonstrated a correlation between ΔLSM and clinical events in patients with MASLD [27]. This is consistent with our finding that a high ΔLSM is a significant risk factor for decompensated cirrhosis and liver cancer; however, we found that overall mortality was also significantly correlated with ΔLSM. The reason for this discrepancy in results is unclear; accordingly, a larger prospective study on the usefulness of serial LSM in both MRE and VCTE is warranted. Advantages of MRE over VCTE include the ability to evaluate liver morphology by imaging, less susceptibility to heterogeneity of liver fibrosis, applicability to patients with ascites and marked obesity, and less susceptibility to the skill of the operator [17, 28].

This study had several limitations. First, this study included patients diagnosed with cirrhosis without liver biopsy. Although liver biopsy has several problems, including sampling error, intra-/inter-observer error, invasiveness, and cost, it remains the gold standard for the diagnosis of cirrhosis. Second, the definition of decompensated cirrhosis follows previous reports, but there is ambiguity in the diagnosis of decompensated cirrhosis, including the presence of ascites. Third, this was a retrospective analysis. Fourth, patient enrollment and data collection were not conducted in accordance with established protocols, which may have led to data heterogeneity and selection bias. Fifth, this study was conducted in a single tertiary care center and only included Japanese patients. Future studies should investigate the generalizability of the results to other populations. Serial MRE measurements have only recently become possible, and further studies are needed.

In conclusion, this study demonstrated that ΔLSM can be used to predict the development of cirrhosis in patients with non-cirrhotic MASLD. Additionally, the combination of initial and ΔLSM assessments may allow for more detailed risk stratification in patients with MASLD. In patients with MASLD, ΔLSM may help to identify patients at a high risk of clinical events among those previously considered as low risk based on a low baseline LSM.

### Supplementary Information

Below is the link to the electronic supplementary material.

Supplementary file 1 (DOCX 39 KB)

Supplementary file 2 (TIF 631 KB)

Supplementary file 3 (TIF 745 KB)

Supplementary file 4 (TIF 522 KB)

## Data Availability

Data can be available upon reasonable request to the corresponding author.
