# Supplementary material for: Prediction of outcomes in patients with metabolic dysfunction-associated steatotic liver disease based on initial measurements and subsequent changes in magnetic resonance elastography
Source: J Gastroenterol. 2023 Oct 16;59(1):56–65. doi: 10.1007/s00535-023-02049-9 (PMC10764489; doi:10.1007/s00535-023-02049-9)
Supplement: Supplementary file 1 — (DOCX 39 KB) [file 535_2023_2049_MOESM1_ESM.docx]

**Supplementary material**

**Prediction of outcomes in patients with metabolic dysfunction-associated steatotic liver disease based on initial measurements and subsequent changes in magnetic resonance elastography**

*Journal of Gastroenterology*

Takashi Kobayashi, Michihiro Iwaki, Asako Nogami, Nobuyoshi Kawamura, Yasushi Honda, Yuji Ogawa, Kento Imajo, Masato Yoneda*, Satoru Saito, and Atsushi Nakajima

*Correspondence: Masato Yoneda, Department of Gastroenterology and Hepatology, Yokohama City University Graduate School of Medicine, 3-9 Fukuura, Kanazawa-ku, Yokohama 236-0004, Japan; E-mail: [yoneda@yokohama-cu.ac.jp](mailto:yoneda@yokohama-cu.ac.jp)

**Supplementary Fig. 1.** Cumulative incidence of overall clinical events based on LSM in patients with metabolic dysfunction-associated steatotic liver disease (MASLD)

(a) Comparison of patients divided into five groups (Groups 0–4) corresponding to each fibrosis stage (fibrosis stage 0–4) based on the initial LSM. (b) Comparison of progressors vs. non-progressors based on ΔLSM.

LSM, liver stiffness measurement by magnetic resonance elastography; ΔLSM, difference between two liver stiffness measurements by magnetic resonance elastography

**Supplementary Fig. 2.** Cumulative incidence of clinical events in progressors vs. non-progressors (stable/regression) based on ΔLSM in patients without cirrhosis (n=386)

(a) Liver-related events; (b) decompensated cirrhosis; (c) hepatocellular carcinoma; (d) all-cause mortality; (e) extrahepatic malignancies; (f) cardiovascular disease.

ΔLSM: difference between two liver stiffness measurements by magnetic resonance elastography

**Supplementary Fig. 3.** Cumulative incidence of cirrhosis development in progressors vs. non-progressors (stable/regression) based on ΔLSM in patients with metabolic dysfunction-associated steatotic liver disease (MASLD) without cirrhosis at baseline (n = 386)

ΔLSM: difference between two liver stiffness measurements by magnetic resonance elastography

S**upplementary Table 1. Extrahepatic malignancies in patients with metabolic dysfunction-associated steatotic liver disease (n=405)**

| Cancer type | Number |
| --- | --- |
| Colorectal cancer | 7 |
| Breast cancer | 2 |
| Gastric cancer | 2 |
| Pancreatic cancer | 2 |
| Lung cancer | 2 |
| Uterine cancer | 1 |
| Prostate cancer | 1 |
| Tongue cancer | 1 |
| Cholangiocarcinoma | 1 |
| Laryngeal cancer | 1 |
| Duodenal cancer | 1 |
| Malignant lymphoma | 1 |
| Total | 22 |

S**upplementary Table 2. Relative risk of clinical outcomes according to the initial LSM in patients with metabolic dysfunction-associated steatotic liver disease (n=405)**

| Event | Initial LSM group | HR | 95% CI | *p* |
| --- | --- | --- | --- | --- |
| Liver-related events | 0 | 1 (reference) |  |  |
|  | 1 | 0.26 | 0.003–21.85 | 0.549 |
|  | 2 | 4.60 | 0.41–51.1 | 0.214 |
|  | 3 | 10.48 | 1.55–70.68 | 0.006 |
|  | 4 | 37.01 | 8.67–157.9 | <0.001 |
| Decompensated cirrhosis | 0 | 1 (reference) |  |  |
|  | 1 | 0.26 | 0.003–21.85 | 0.549 |
|  | 2 | 0.22 | 0.003–14.46 | 0.482 |
|  | 3 | 4.81 | 0.42–54.28 | 0.203 |
|  | 4 | 24.84 | 4.51–137.0 | <0.001 |
| HCC | 0 | 1 (reference) |  |  |
|  | 1 | NA | NA | NA |
|  | 2 | 19.94 | 1.06–376.7 | 0.046 |
|  | 3 | 34.53 | 1.59–750.5 | 0.024 |
|  | 4 | 60.29 | 6.15–591.1 | <0.001 |
| All-cause mortality | 0 | 1 (reference) |  |  |
|  | 1 | NA | NA | NA |
|  | 2 | 21.00 | 0.32–1363 | 0.153 |
|  | 3 | 33.02 | 3.78–288.9 | 0.003 |
|  | 4 | 45.33 | 6.17–333.0 | <0.001 |
| Extrahepatic malignancies | 0 | 1 (reference) |  |  |
|  | 1 | 0.255 | 0.047–1.36 | 0.110 |
|  | 2 | 1.15 | 0.328–4.035 | 0.826 |
|  | 3 | 1.24 | 0.341–4.507 | 0.744 |
|  | 4 | 3.66 | 1.098–12.19 | 0.035 |
| Cardiovascular disease | 0 | 1 (reference) |  |  |
|  | 1 | 34.94 | 0.447–2730 | 0.110 |
|  | 2 | 18.20 | 0.294–1125 | 0.168 |
|  | 3 | 35.42 | 1.62–775.1 | 0.024 |
|  | 4 | 64.88 | 2.52–1668 | 0.012 |
| Overall events | 0 | 1 (reference) |  |  |
|  | 1 | 0.45 | 0.10–2.00 | 0.297 |
|  | 2 | 1.87 | 0.64–5.51 | 0.254 |
|  | 3 | 3.97 | 1.47–10.71 | 0.007 |
|  | 4 | 13.48 | 5.28–34.43 | <0.001 |

The initial LSM group corresponds to liver fibrosis stages 0–4.

CI, confidence interval; HCC, hepatocellular carcinoma; HR, hazard ratio; LSM, liver stiffness measurement; NA, not applicable

**Supplementary Table 3. Cumulative probability of outcomes in each group according to ΔLSM (n=405)**

| Event | Groups divided by ΔLSM | Events, n | Cumulative, %＊ | *p* |
| --- | --- | --- | --- | --- |
| Liver-related events | Regressor/Stable | 8/353 | 2.3 | 0.001 |
|  | Progressor | 6/52 | 11.5 |  |
| Decompensated cirrhosis | Regressor/Stable | 4/353 | 1.1 | 0.009 |
|  | Progressor | 5/52 | 9.6 |  |
| Hepatocellular carcinoma | Regressor/Stable | 3/353 | 0.8 | 0.008 |
|  | Progressor | 3/52 | 5.8 |  |
| Overall death | Regressor/Stable | 9/353 | 2.5 | 0.820 |
|  | Progressor | 2/52 | 3.8 |  |
| Extrahepatic malignancies | Regressor/Stable | 13/353 | 3.7 | 0.180 |
|  | Progressor | 4/52 | 7.7 |  |
| Cardiovascular disease | Regressor/Stable | 2/353 | 0.6 | 0.317 |
|  | Progressor | 1/52 | 1.9 |  |
| Overall events | Regressor/Stable | 27/353 | 7.6 | 0.008 |
|  | Progressor | 10/52 | 19.2 |  |

＊Estimated using Kaplan–Meier analysis

Patients were classified as progressors (ΔLSM ≥19%) and non-progressors (ΔLSM <19%) according to the difference between the first and last liver stiffness measurement (ΔLSM).

**Supplementary Table 4. Cumulative probability of outcomes in each group divided by ΔLSM in the cohort with low initial LSM (N = 296)**

| Event | Groups divided by ΔLSM | Events, n | Cumulative, %＊ | P value |
| --- | --- | --- | --- | --- |
| Liver-related events | Regressor/Stable | 1/255 | 0.4 | 0.009 |
|  | Progressor | 2/41 | 4.9 |  |
| Overall events | Regressor/Stable | 4/255 | 1.6 | 0.012 |
|  | Progressor | 6/41 | 14.6 |  |

＊Estimated using Kaplan–Meier analysis.

Patients were classified as progressors (ΔLSM ≥19%) and non-progressors (ΔLSM <19%) according to the difference between the first and last liver stiffness measurement (ΔLSM).

**Supplementary Table 5. Hazard ratios and 95% CIs for outcome of progressors compared with non-progressors in the cohort with low initial LSM (n=296)**

| Event | Hazard ratio | 95% CI of HR | *p* |
| --- | --- | --- | --- |
| Liver-related events | 77.45 | 2.99–2006 | 0.009 |
| Overall events | 10.32 | 1.65–64.37 | 0.012 |

CI, confidence interval; HR, hazard ratio
