# Supplementary figures and images for: Prediction of outcomes in patients with metabolic dysfunction-associated steatotic liver disease based on initial measurements and subsequent changes in magnetic resonance elastography
Source: J Gastroenterol. 2023 Oct 16;59(1):56–65. doi: 10.1007/s00535-023-02049-9 (PMC10764489; doi:10.1007/s00535-023-02049-9)

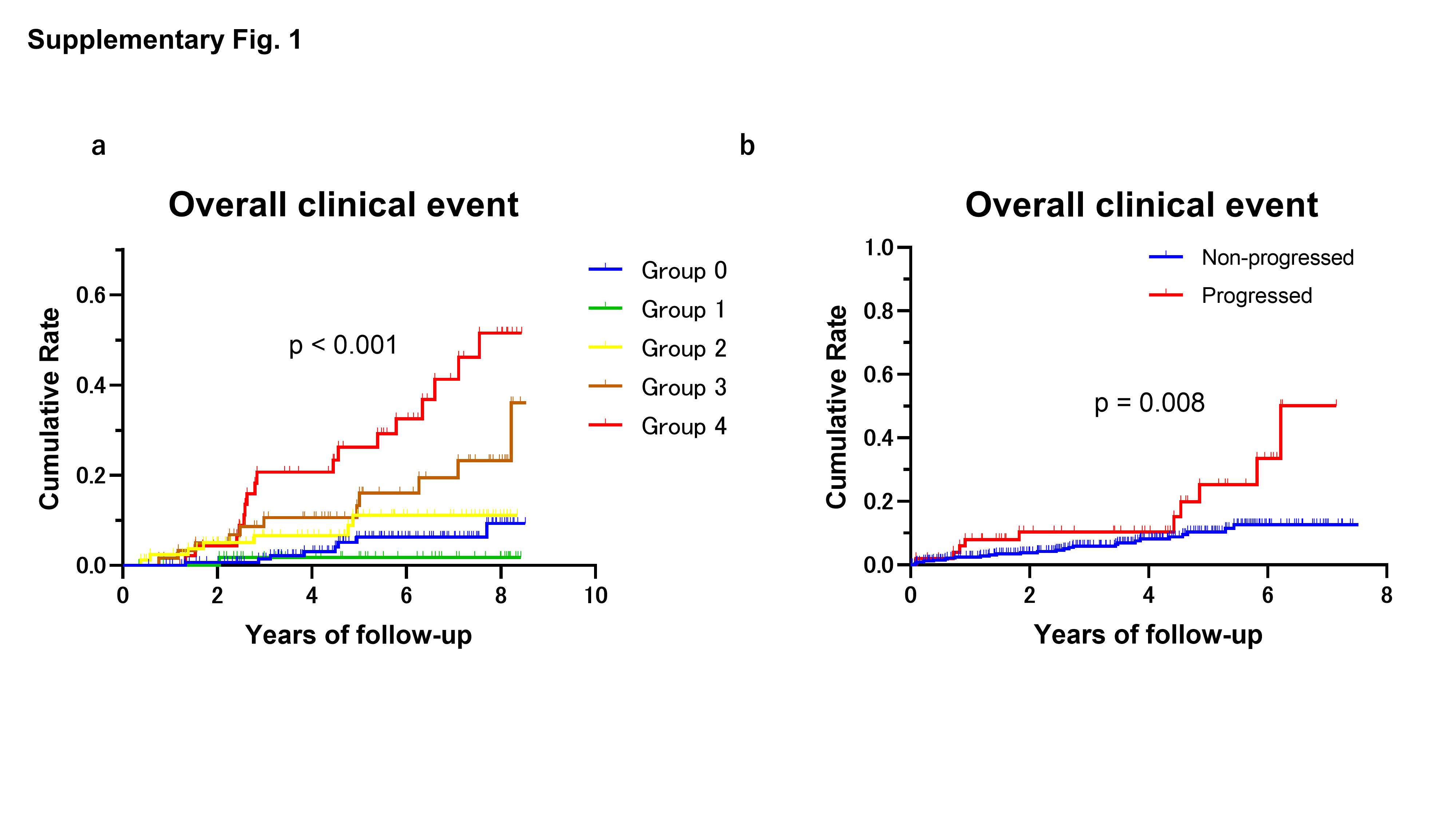

Supplement: Supplementary file 2 — (TIF 631 KB) [file 535_2023_2049_MOESM2_ESM.tif]

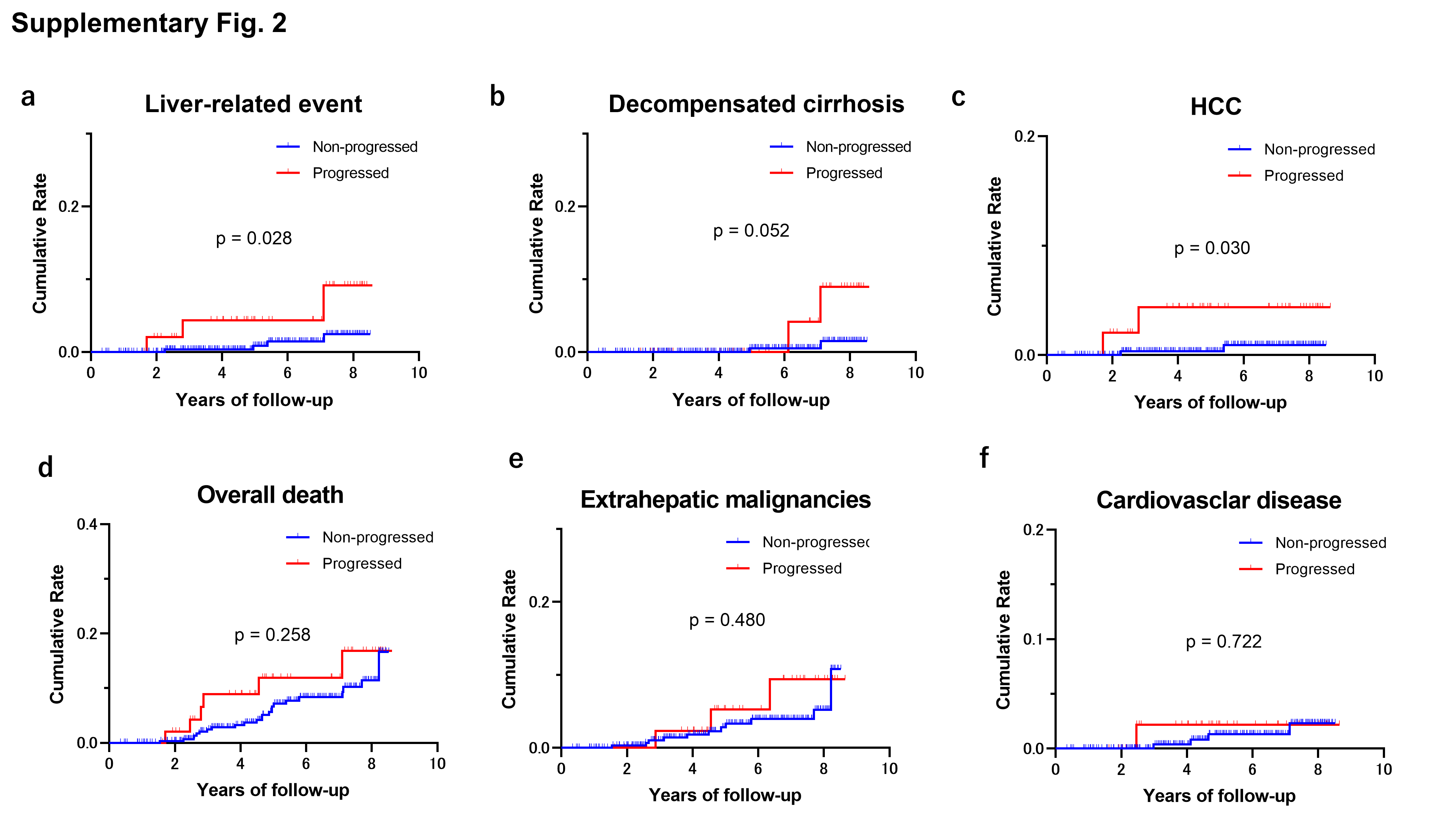

Supplement: Supplementary file 3 — (TIF 745 KB) [file 535_2023_2049_MOESM3_ESM.tif]

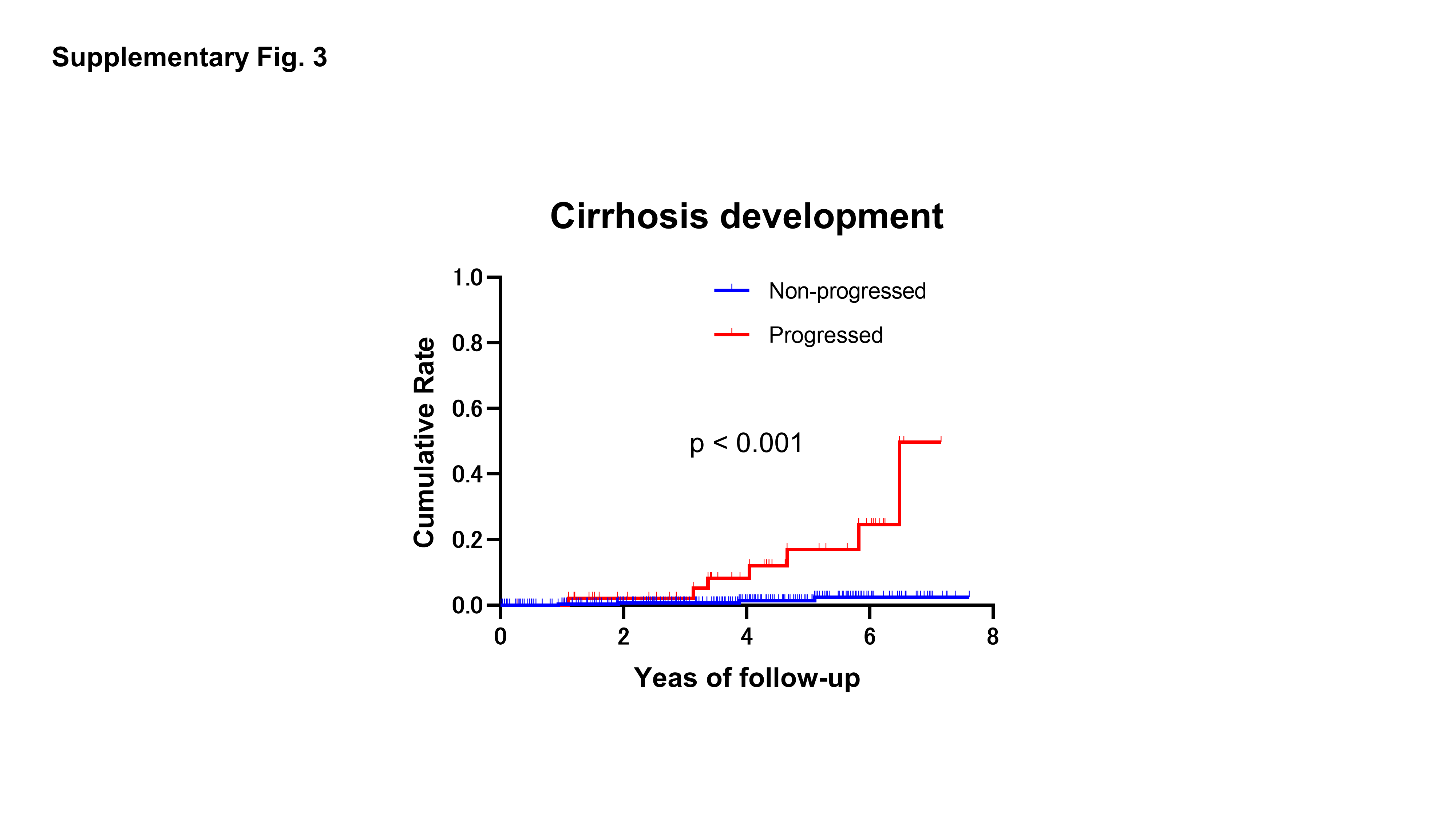

Supplement: Supplementary file 4 — (TIF 522 KB) [file 535_2023_2049_MOESM4_ESM.tif]
